# Optimization of Enterotoxigenic *Escherichia coli* (ETEC) Outer Membrane Vesicles Production and Isolation Method for Vaccination Purposes

**DOI:** 10.3390/microorganisms11082088

**Published:** 2023-08-15

**Authors:** Melibea Berzosa, Alberto Delgado-López, Juan Manuel Irache, Carlos Gamazo

**Affiliations:** 1 Department of Microbiology and Parasitology, Navarra Medical Research Institute (IdiSNA), University of Navarra, 31008 Pamplona, Spain; adlopez@unav.es; 2 Department of Pharmacy and Pharmaceutical Technology, University of Navarra, 31008 Pamplona, Spain; jmirache@unav.es

**Keywords:** Enterotoxigenic *Escherichia coli* (ETEC), outer membrane vesicle (OMV), vaccine, immunogenicity, ultracentrifugation, ultrafiltration

## Abstract

The study addresses Enterotoxigenic *Escherichia coli* (ETEC), a significant concern in low-income countries. Despite its prevalence, there is no licensed vaccine against ETEC. Bacterial vesicle-based vaccines are promising due to their safety and diverse virulence factors. However, cost-effective production requires enhancing vesicle yield while considering altered properties due to isolation methods. The proposed method involves heat treatment and ultrafiltration to recover vesicles from bacterial cultures. Two vesicle types, collected from heat-treated (HT-OMV) or untreated (NT-OMV) cultures, were compared. Vesicles were isolated via ultrafiltration alone (“complete”) or with ultracentrifugation (“sediment”). Preliminary findings suggest complete HT-OMV vesicles are suitable for an ETEC vaccine. They express important proteins (OmpA, OmpX, OmpW) and virulence factors (adhesin TibA). Sized optimally (50–200 nm) for mucosal vaccination, they activate macrophages, inducing marker expression (CD40, MHCII, CD80, CD86) and Th1/Th2 cytokine release (IL-6, MCP-1, TNF-α, IL12p70, IL-10). This study confirms non-toxicity in RAW 264.7 cells and the in vivo ability of complete HT-OMV to generate significant IgG2a/IgG1 serum antibodies. Results suggest promise for a cost-effective ETEC vaccine, requiring further research on in vivo toxicity, pathogen-specific antibody detection, and protective efficacy.

## 1. Introduction

Enterotoxigenic *Escherichia coli* (ETEC) strains belong to one of the most virulent *E. coli* pathotypes. ETEC infections are responsible for millions of diarrhea illness cases, which mainly affect children under the age of five in low- and middle-income countries [1]. Moreover, this disease is associated with sequelae such as childhood growth stunting and undernourishment. Therefore, it is believed that mortality caused by ETEC infections are underestimated [1,2]. Furthermore, ETEC is the most common cause of acute diarrhea among international travelers to high-risk endemic regions [3]. ETEC exhibits remarkable genetic plasticity and can express a wide variety of pathogen-specific virulence factors (e.g., heat-labile enterotoxin (LT), heat-stable enterotoxin (ST), and colonization factors (CFs)) which vary across regions and populations and over time, thereby frustrating efforts to identify an effective vaccine [4,5,6,7]. Thus, ETEC vaccine development is a World Health Organization (WHO) priority [8]. Different vaccine approaches are currently being pursued, including those based in outer membrane vesicles (OMV) [9].

NT-OMVs are vesicles released from the outer membrane of Gram-negative bacteria which contain a variety of cellular components including lipopolysaccharides (LPS), lipoproteins, and other virulence factors [10,11]. OMVs offer at least three main advantages: (i) stimulation of both cellular and antibody-mediated immune responses; (ii) suitability for the design of mucosal vaccines; and (iii) good safety profile [12]. Despite these advantages, OMV-based vaccines present an important challenge related to the low yield of the current processes for obtaining these vesicles from bacterial cultures [13]. Basically, the method for the production and purification of native OMV relies on: (i) culture and incubation (the vesicles are naturally released from the bacteria); (ii) removal of intact bacteria by centrifugation and filtration; (iii) OMV isolation from the filtered supernatant; and (iv) OMV purification by ultracentrifugation or exclusion chromatography [14]. In order to minimize this problem and, thus, increase the efficacy of the preparative process, different approaches have been proposed, including the use of stress factors such as increased temperature [15], ultraviolet radiation [16], or the addition of surfactants to the culture media [17]. In this way, for the development of the licensed NT-OMV-based vaccine against Neisseria meningitidis C (Bexsero^®^), a deoxycholate or sodium dodecyl sulfate treatment has been employed [18,19]. Vesicle-overproducing strain mutants have also been tested [20,21].

Our goal was to evaluate the effect of heat treatment on bacterial cultures of ETEC to induce a higher release of vesicles, followed by OMV isolation by ultrafiltration, avoiding the expensive ultracentrifugation step. Thus, in this work, we compared vesicles collected after bacterial cultures heat treatment (named HT-OMV) with native vesicles (named NT-OMV). Furthermore, we compared NT-OMV and HT-OMV isolated by ultrafiltration (named “complete”) with the ones obtained by ultrafiltration and ultracentrifugation (named “sediment”). We determined bacterial vesicle yield, composition, antigenicity, cytotoxicity, and their immunogenicity in BALB/c mice. This study provides an improvement of the bacterial vesicle production and isolation method to use them as a vaccine antigenic complex, contributing to knowledge for the development of a broad-protective ETEC vaccine.

## 2. Materials and Methods

### 2.1. Animal Ethics Statement

The protocol for animal experiments was approved by the Experimental Animal Ethical Committee of the University of Navarra (approval number: 027-20).

### 2.2. Bacterial Strain and Cell Line

The Enterotoxigenic *E. coli* O78:H11 reference strain was purchased from the American Type Culture Collection (ATCC 35401). The RAW 264.7 murine macrophage cell line was purchased from the American Type Culture Collection (Rockville, MD, USA). Finally, the renal epithelial cells HEK-293 modified to express only the Toll Like Receptor (TLR)- 4 were developed and kindly provided by Dr. Andra B. Schromm from the Division of Immunobiophysics, Priority Area Infections, Research Center Borstel, Leibniz Lung Center, Borstel, Germany.

### 2.3. Vesicles Production and Purification

**Figure 1:**
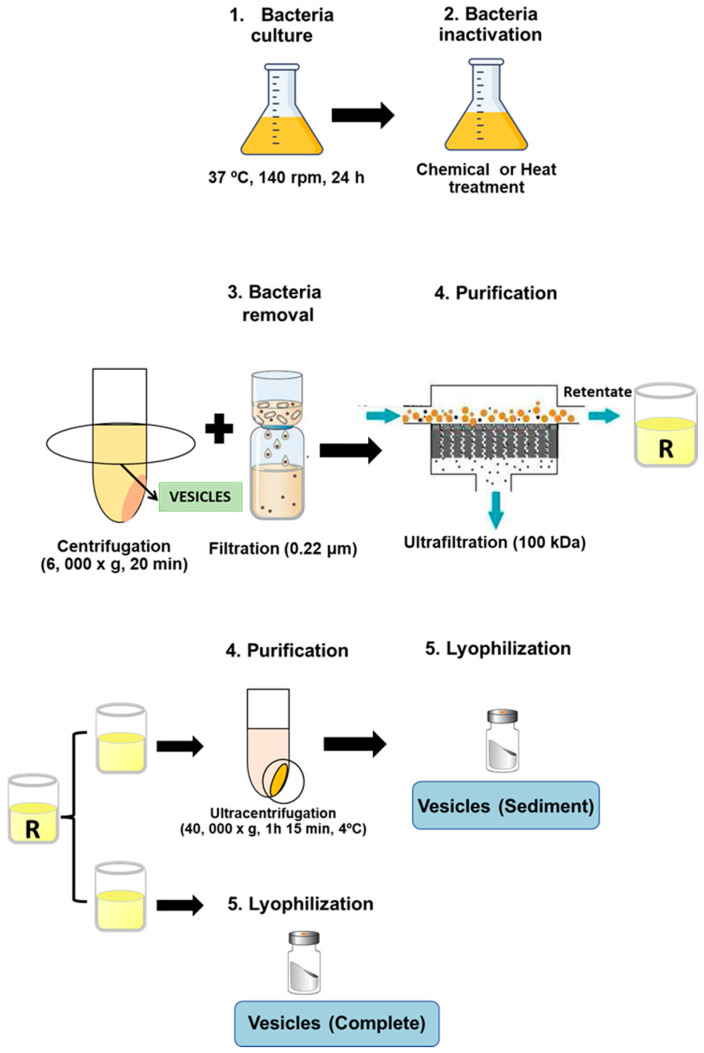
Diagram showing bacterial vesicle production and isolation. Cultures of the reference strain ETEC H10407 (ATCC 35401) were inactivated by chemical with either 6 mM binary ethylenimine, 0.06% formaldehyde, and 50 mM ethylenediaminetetraacetic acid (named NT-OMV) or by heat treatment steam flow at 100 °C, 15 min (named HT-OMV). Then, cultures were centrifuged and filtered (0.22 μm) to remove bacteria. The obtained supernatant was ultrafiltered (100 kDa) and the retenant was divided into two equal aliquots. The first one was lyophilized (complete vesicles). The second one was ultracentrifuged (40,000× *g*, 1 h 15 min, 4 °C), and lyophilized (sediment vesicles).

Cryobeads (Microkit laboratories, Madrid, Spain) conserved at −80 °C containing ETEC strains were incubated in TSA plates (Biomerieux, Madrid, Spain) at 37 °C, 24 h to prepare a bacterial suspension (optical density at 600 nm of 0.125) to inoculate 500 mL TSB (Biomerieux, Spain) and incubate it at 37 °C, 140 rpm, 24 h. Then, bacterial cultures were inactivated by chemical treatment with either binary ethylenimine (6 mM, Sigma-Aldrich Madrid, Spain), formaldehyde (0.06%, Panreac), and ethylenediaminetetraacetic acid (50 mM, Sigma-Aldrich) (BEI-FA-EDTA), or by a heat treatment steam flow at 100 °C, 15 min. Thus, outer membrane vesicles NT-OMV were obtained from the bacteria inactivated with BEI-FA-EDTA, whereas HT-OMV were obtained from the heat-treated cultures. After that, bacteria were removed from cultures by centrifugation at 6000× *g*, 20 min and filtration through 0.22 μm pore size filters (Corning, New York, USA), and vesicles present in the supernatant of the cultures were collected by tangential ultrafiltration (molecular weight cut-off of 100 kDa). Retentate was divided into two equal aliquots, the first one was directly lyophilized (complete vesicles), whereas the other one was ultracentrifuged (40,000× *g*, 1 h 15 min, 4 °C) and lyophilized (sediment vesicles). Consequently, four extracts were obtained depending on their method of production and purification: (i) ultracentrifuged NT-OMV (named “sediment NT-OMV”), (ii) non-ultracentrifuged NT-OMV (named “complete NT-OMV”), (iii) ultracentrifuged HT-OMV (named “sediment HT-OMV”), and (iv) non-ultracentrifuged HT-OMV (named “complete HT-OMV”), as depicted in Figure 1. Bacterial vesicle yield was obtained from final product amount (dry weight) and was referred to the corresponding cellular pellet obtained after first centrifugation (wet cell weight).

### 2.4. Bacterial Vesicles Size Analysis

Vesicles of each extract were dispersed in deionized water and their size was determined by photon correlation spectroscopy (PCS) at 25 °C with a scattering angle of 90° using a Zetasizer analyzer system (Malvern^®^ Instruments, Malvern, UK). For aggregation studies, vesicle size was determined after treatment with PBS-Tween (10 mM sodium phosphate, 0.15 M NaCl, 0.05% Tween-20, pH 7.5) and further sonication at 50 W, 2 min.

### 2.5. Quantitative Analysis of the Protein Content

Total protein content was determined by the Lowry method using bovine serum albumin as standard [22]. To compare the protein profile of each sample, SDS-PAGE was used under denaturalizing conditions using samples previously treated at 100 °C, 10 min in Tris-HCl 62.5 mM, pH 6.8; 10% glycerol; 2% SDS, 5% β-mercaptoethanol and bromophenol blue. The electrophoresis was performed in polyacrylamide gels (12%) (Criterion XT, Bio Rad Laboratories, Hercules, CA, USA) that were further stained with Coomassie brilliant blue (Bio Rad). The apparent molecular weight of the proteins was determined by comparing standards with a known molecular weight (Amershan Pharmacia Biotech, Amersham, UK). Then, to identify bacterial vesicle (NT-OMV sediment and complete; HT-OMV sediment and complete fractions) proteins, mass spectrometry (MS)-based proteome analysis was performed. Samples were homogenized in lysis buffer (7 M urea, 2 M thiourea, 50 mM DTT) and protein digestion was performed as previously described [23]. MS analysis of the resulting peptides were performed in a Sciex 5600 Triple-TOF system (Sciex, Framingham, MA, USA), as described elsewhere [24]. The MS/MS data acquisition was performed using Analyst 1.7.1 (Sciex) and spectra files were processed through Protein Pilot Software (v 5.0.1-Sciex) using ParagonTM algorithm (v 5.0.1) for database search [25], ProgroupTM for data grouping, and compared against the concatenated target-decoy UniProt proteome database (ETEC). False discovery rate was evaluated using a non-linear fitting method [26] and displayed results were those reporting a 1% global false discovery rate or lower. The peptide quantitation was performed using the Progenesis LC-MS software v3.0 [23]. Proteins were quantified with at least two unique peptides.

### 2.6. Qualitative and Quantitative Analysis of the LPS

LPS quantification was performed by the Purpald method [27], using 3-deoxy-α-D-mannooctulosonic acid (KDO) as standard. Moreover, the LPS pattern was analyzed by SDS-PAGE as explained before (see Section 2.5) and LPS molecules were revealed using ammoniacal silver following the Tsai and Frasch method [28].

### 2.7. Polyclonal Antibodies Production

To produce specific polyclonal antibodies against NT-OMV and HT-OMV sediment, New Zealand White rabbits (Envigo, Indianapolis, IN, USA) were immunized with 1 mg of the corresponding antigen in 0.2 mL of PBS, intramuscularly. Blood samples were collected before immunization and weekly until four weeks post-immunization. The presence of specific antibodies against bacterial vesicles in serum were determined by immunoblotting at week 0, 1, 2, 3, and 4 post-immunization. Immunoblotting was performed using nitrocellulose membranes (Whatman Protran^®^; Merk kGaA, Darmstadt, Germany, pore size 0.45 µm) and a semidry blotting system at 0.8 mA/cm2 for 30 min (Trans-Blot^®^ SD Transfer Cell, BIO-RAD, Hercules, CA, USA). After that, protein-binding sites were blocked with PBS with 5% skimmed milk, overnight, at room temperature. Then, the membranes were washed 3 times with PBS-Tween and then incubated (4 h at 4 °C) with sera from hyperimmunized rabbits collected at week 0, 1, 2, 3, and 4 post-immunization. The membranes were washed 3 times with PBS-Tween and then peroxidase-conjugated secondary antibody (anti-IgGFc,) was added for 60 min at room temperature. Finally, membranes were washed with PBS-Tween and the antibody-antigen complexes were visualized after addition of a substrate/chromogen solution (H_2_O_2_/chloro α-naphthol).

### 2.8. Immunoblotting

The antigenicity of the proteins present in the vesicle types analyzed in this work was determined by immunoblotting as described in Section 2.5. The sera from hyperimmunized rabbits with HT-OMV or NT-OMV isolated from ETEC H10407 were used at 1:160 dilution in PBS.

### 2.9. Cellular Assays

#### 2.9.1. HEK293-TLR4 Cell Stimulation Assay

To determine vesicles’ capacity to activate cells through TLR4, HEK293 cells expressing only TLR4 were used. Briefly, HEK293-TLR4 cells were incubated in DMEN medium (Dubecco’s Modified Eagle’s Medium) supplemented with 2 mM L-Glutamine (Gibco^®^, Madrid, Spain), 10% Fetal Bovine Serum (Gibco^®^) and the following antibiotics: penicillin (10,000 units/mL), streptomycin (10,000 g/mL) (Gibco^®^), hygromycin (400 U/mL), and geneticin G418 (0.5 mg/mL). Cells were maintained at sub-confluence in a 5% CO_2_ humidified atmosphere at 37 °C. Then, 2 × 10^5^ cells were added to each well of a 24-well plate (Corning) and incubated for 24 h. After cell proliferation, they were treated with 1 µg/mL of NT-OMV or HT-OMV complete or sediment during 24 h. Culture supernatants were analyzed by ELISA to detect the presence of IL-8. 

#### 2.9.2. Cell Viability Assay

RAW 264.7 cells were maintained in DMEN medium supplemented with 2 mM L-Glutamine, 10% Fetal Bovine Serum and penicillin (10,000 units/mL) streptomycin (10,000 g/mL) at sub-confluence in 5% CO_2_ humidified atmosphere at 37 °C. Then, 5 × 10^4^ cells were seeded in each well of a microplate (Agilent Technologies) and incubated for 24 h to monitor cell proliferation. Following the cell proliferation step, cells were treated with different concentrations of HT-OMV (sediment) (64 μg/mL to 1 μg/mL) for 72 h to determine the adequate vesicle concentration to use in a final comparative experiment. In this assay, after cell proliferation, 1 μg/mL of either NT-OMV or HT-OMV (sediment or complete) was added and incubated for 72 h. Cell proliferation was monitored using the Real-Time Analysis System (xCELLigence RTCA, Agilent technologies, Santa Clara, CA, USA). Non-treated cells and cells treated with amphotericin B (12.5 mg/mL, Gibco^®^) were used as control of viable or dead cells, respectively. 

#### 2.9.3. Macrophage Stimulation Assay

To investigate the effect of the vesicles on macrophage activation, specific activation markers and cytokines were detected in vitro. RAW 264.7 cells were incubated in 24-well plates (Falcon^®^, Fisher Scientific, Madrid, Spain) at 37 °C, 5% CO_2_, for 12 h. After that, cells were treated with 1 µg/mL of the corresponding extract and incubated for 24 h, 37 °C, 5% CO_2_. Then, supernatants were removed to determine the presence of the following cytokines: IL-6, MCP-1, IL-10, TNF-α, and IL12p70 using a BD™ Cytometric Bead Array (CBA) mouse inflammation kit. Moreover, cells were labeled with the corresponding specific monoclonal antibodies conjugated with a fluorochrome: MHCII (Anti-MHC class II-FITC mouse, MACS), CD40 (FITC anti-mouse CD40, Biolegend, San Diego, CA, USA), CD80 (PE anti-rat CD80, Biolegend), and CD86 (PE anti-mouse, Biolegend). 

### 2.10. Mice Immunization and Specific Antibody Response

To investigate the immunogenicity of the bacterial vesicles, female BALB/c mice (20 ± 1 g) (Envigo, Indianapolis, IN, USA) were randomized in groups of 5 animals. Mice were immunized with a nasal dose of 25 µg of NT-OMV or HT-OMV, complete or sediment obtained from ETEC H10407. Non-immunized mice were used as a control. Blood samples were collected before immunization and weekly until four weeks post-immunization. Specific IgG1 and IgG2a antibodies against bacterial vesicles in serum were determined by indirect ELISA at week 0, 1, 2, 3, and 4 post-immunization.

Briefly, 96-well plates (MaxiSorp; Nunc, Fisher Scientific, Madrid, Spain) were coated with 100 µL of the corresponding vesicle type at a concentration of 10 µg/mL in a coating buffer (60 mM carbonate buffer, pH 9.6). Unspecific binding sites were blocked with 3% bovine serum albumin (BSA) in PBS for 1 h at room temperature. Pools of sera from the immunized mice were diluted 1:100 with 1% BSA in PBS and incubated for 4 h at room temperature. After five washes with PBS-Tween buffer, anti-mouse IgG2a (Sigma-Aldrich) or anti-mouse IgG1 (Nordic) conjugated antibodies were added and incubated for 1 h at room temperature. The detection was carried out by incubating the sample with H_2_O_2_-ABTS™ substrate-chromogen for 15 min at room temperature. Absorbance was measured with a microplate reader (Tecan, Männedorf, Switzerland) at a wavelength of 405 nm.

### 2.11. Statistical Analysis

After the Shapiro–Wilk normality test, statistical analyses between five groups were performed by one-way ANOVA test (parametric) followed by Dunnett’s multiple comparisons tests. GraphPad Prism7^®^ software (San Diego, CA, USA) was used to perform the analysis. Significance was established for *p* values lower than 0.05 and represented by * (*p* < 0.05), ** (*p* < 0.01), *** (*p* < 0.001), and **** (*p* < 0.0001). All values are expressed as mean ± standard deviation (SD). 

## 3. Results

### 3.1. Yield

The primary aim of this study was to compare the resulting yield of NT-OMV or HT-OMV complete or sediment. Heat-treated cultures showed a yield in HT-OMV 5-fold higher than non-treated cultures (NT-OMV) (1.7 μg/mg vs. 0.3 μg/mg). Moreover, non-ultracentrifugated (complete) samples presented a yield 9-fold higher compared to NT-OMV ultracentrifugated (sediment) (4.6 μg/mg), and 3-fold higher compared to sediment HT-OMV (2.7 μg/mg). 

### 3.2. Bacterial Vesicles Characterization

#### 3.2.1. Size

The vesicle’s size affects their cellular uptake mechanism and, therefore, their fate and intracellular effects. The four samples showed a similar percentage of vesicles (~40%) ranging 50–300 nm. Complete samples presented a higher percentage of small vesicles (<50 nm), whereas larger vesicles (>300 nm) were found in sediment extracts (Table 1). To verify if this last population (>300 nm) were individual particles or aggregates, samples were resuspended in PBS-Tween and sonicated. The percentage of vesicles > 300 nm after treatment decreased, in correlation with the increased number of smaller vesicles (<50 nm or 50–300 nm), suggesting the presence of aggregates in those large vesicles (>300 nm diameter) (Table 1).

#### 3.2.2. Quantitative and Qualitative Protein Analysis

Protein content among sediment samples was similar to complete samples, consisting of 16.8 ± 0.0% vs. 17.4 ± 0.0% in NT-OMV and 22.5% ± 0.0% vs. 23.4 ± 0.1% in HT-OMV (sediment vs. complete, respectively).

Proteomic analysis showed the presence of (i) 1681 proteins in NT-OMV (sediment), (ii) 1696 proteins in NT-OMV (complete), (iii) 890 proteins in HT-OMV (sediment), and (iv) 1366 proteins in HT-OMV (complete); with at least 6 unique peptides. Among them, immunodominant proteins commonly expressed by Enterobacteriaceae bacteria were identified, for instance, outer membrane proteins (Omp: A, C, X, W, F), the antigen 43, Skp, YghJ, or lipoproteins. Flagellin was also present in the four bacterial vesicle extracts [NT-OMV and HT-OMV (sediment and complete fractions)], showing similar quantification values (Table 2). 

Furthermore, proteome analysis demonstrated the presence of important ETEC immunogenic virulence factors, such as colonization factor I (CFA/I), the heat-labile enterotoxin (LT), and non-classical factors (EtpA, EatA and TibA), that are crucial in a vaccine strategy (Table 3). CFA/I and non-classical virulence factors were found in similar quantities in the four bacterial vesicles: NT-OMV and HT-OMV (sediment and complete fractions). Interestingly, regarding LT enterotoxin (toxin which provokes the acute diarrhea), while there are similar levels of the binding subunit B of the toxin, the enzymatic A subunit is decreased in HT-OMV vesicles, being zero in the HT-OMV complete fraction (Table 3).

#### 3.2.3. Quantitative and Qualitative LPS Analysis

Data from LPS quantification indicated that samples analyzed in this study had similar LPS levels (0.81 mM and 0.87 mM in NT-OMV; 0.66 mM and 0.63 mM; sediment and complete, respectively). Moreover, the LPS pattern was determined by SDS-PAGE (Figure S1), showing no differences among samples and the LPS pattern detected was as previously described [29].

#### 3.2.4. Antigenicity

**Figure 2:**
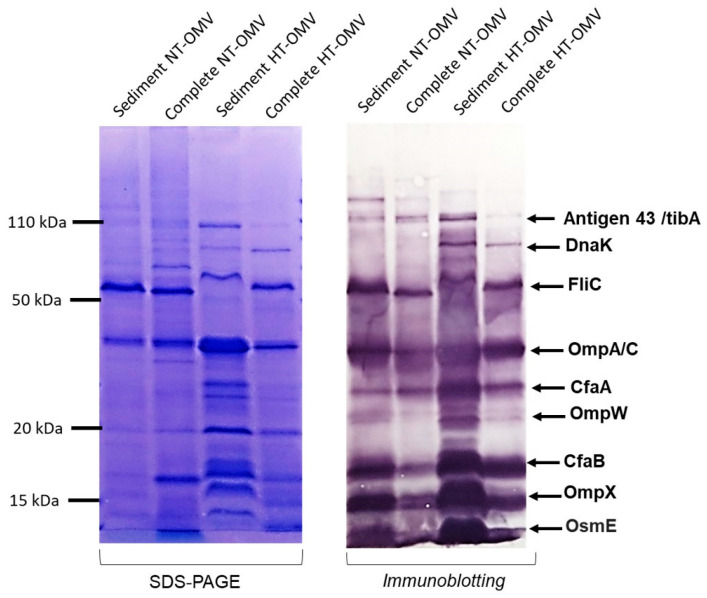
Antigenicity of the outer membrane vesicle naturally released (NT-OMV) or heat-treated (HT-OMV) from Enterotoxigenic *Escherichia coli* (ETEC) (ATCC 35401) and collected by ultrafiltration (complete) or ultracentrifugation (sediment). SDS-PAGE with Coomassie blue staining of each sample is shown on the left indicating the protein profile of the extracts. On the right, immunoblotting of the samples using sera from hyperimmunized rabbits with NT-OMV or HT-OMV obtained from ETEC strains is indicated. Molecular weight markers are indicated on the left in kDa and the position of the most abundant proteins detected by proteomic studies on the right.

Bacterial vesicle antigenicity can be altered depending on the production and isolation method used. Thus, to evaluate it, immunoblotting was performed using sera from hyperimmunized rabbits with NT-OMV and HT-OMV from ETEC H10407. None of the suggested methods yielded noticeable changes in the antigenicity of the vesicles (Figure 2). 

### 3.3. Evaluation of In Vitro Biological Activities of the Samples

#### 3.3.1. Activation of HEK293 Cells Expressing TLR4

LPS is both an immunostimulant and endotoxic structure at the cellular level. LPS-TLR4 interaction induces a pro-inflammatory response resulting in the release of pro-inflammatory cytokines, such as IL-8. To investigate the capacity of the extracts analyzed to activate HEK293 cells through the TLR4 pathway, HEK293 cells expressing TLR4 (HEK293/TLR4) were incubated with NT-OMV or HT-OMV complete or sediment and the level of IL-8 produced was quantified by ELISA.

**Figure 3:**
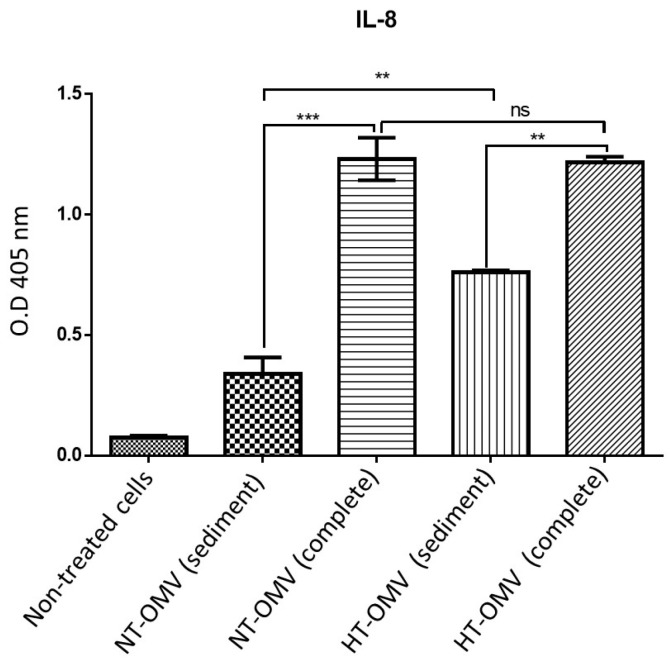
HEK293 expressing TLR4 stimulation with outer membrane vesicle naturally released (NT-OMV) or heat-treated (HT-OMV) from Enterotoxigenic *Escherichia coli* (ETEC) (ATCC 35401) and collected by ultrafiltration (complete) or ultracentrifugation (sediment). HEK293/TLR4 were stimulated with 1 μg/mL of each vesicle type. To determine their activation, the presence of IL-8 was determined in the cell’s supernatants by ELISA (O.D 405 nm). (Not significant (ns) *p* > 0.005; ** *p* < 0.001; *** *p* < 0.0001 vs. non-treated cells). Error bars represent standard deviation (*n* = 3).

HEK293/TLR4 cells were incubated with 1 μg/mL of each vesicle type analyzed in this study at 37 °C, 5% CO_2_, for 24 h. Results indicated that sediment vesicles induced significantly lower levels of IL-8 compared to complete samples. Moreover, no differences between complete vesicles were observed, whereas sediment HT-OMV showed significantly higher IL-8 levels with respect to sediment NT-OMV (Figure 3).

#### 3.3.2. Cell Viability Assays

**Figure 4:**
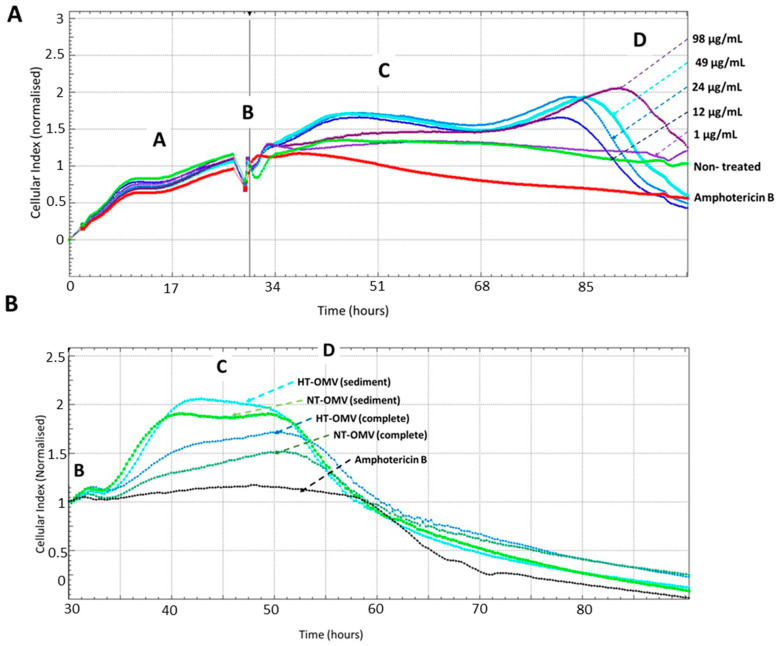
Real-Time analysis of RAW 264.7 macrophages viability after their incubation with different concentrations of outer membrane vesicles heat treated (HT) from Enterotoxigenic *Escherichia coli* (ETEC) (ATCC 35401) collected by ultracentrifugation (sediment) to determine the adequate vesicle concentration to use in a comparative final experiment (**A**) or with outer membrane vesicle naturally released (NT-OMV) or heat treated (HT-OMV) from Enterotoxigenic *Escherichia coli* (ETEC) (ATCC 35401) and collected by ultrafiltration (complete) or ultracentrifugation (sediment) to compare the toxicity of all vesicles analyzed in this study (**B**) (*n* = 3). Macrophages treated with amphotericin B (12.5 mg/mL) were used as cell death control and untreated cells were used as negative control. The cellular toxicity was determined using the Real-Time analysis system (RTCA), which employs disposables E-Plates 16 glass bottom coated with gold microelectrodes where cells are attached. The real-time electrical impedance is interpreted by the software as the Cell Index (CI). As cells become attached to the electrodes, CI values increase proportionally, stablishing the different cellular phases indicated: proliferation cellular (A), extract addition (B), cellular confluence (C), and cellular death (D).

An effective vaccine should not only induce a protective immune response but also be safe. For this reason, the toxicity of NT-OMV and HT-OMV vesicles collected by ultrafiltration (complete) or ultracentrifugation (sediment) on RAW 264.7 macrophages was determined by monitoring cell viability using the Real-Time Cell Analysis (RTCA) system. To determine the adequate vesicle concentration to use in a final comparative experiment, cells were treated with different concentrations of HT-OMV (sediment) (64 μg/mL to 1 μg/mL), selecting 1 μg/mL. Macrophages treated with amphotericin B were used as cell death control and untreated cells were used as negative control. Disposables E-Plates 16 (16-well plates with a glass bottom coated with gold microelectrodes) were utilized for this purpose. The real-time electrical impedance is interpreted by the software as the Cell Index (CI). As cells become attached to the electrodes, CI values increase proportionally. Consequently, the growth curves of the RAW cells (adhesion, proliferation, and stationary phase; see Figure 4) were established. The different cellular phases are indicated as follows: proliferation phase (A), extract addition (B), cellular confluence (C), and cellular death (D).

Data from the dose-response experiment indicated that there was a negative correlation between HT-OMV concentration and cell viability, since the number of cells attached to the bottom of the well decreased when the HT-OMV quantity increased. Furthermore, when 264.7 RAW cells were treated with 1 μg/mL of sediment HT-OMV, these cells presented a similar behavior to that in the untreated cells (Figure 4A). Thus, the comparative study with the four vesicle types analyzed in this work was performed by incubating 264.7 RAW cells with 1 μg/mL of either NT-OMV or HT-OMV (sediment or complete) at 37 °C, 5% CO_2_, for 24 h. No significant differences were found between the samples and no cytotoxicity was detected for 48 h (Figure 4B). 

#### 3.3.3. Phagocytosis

**Figure 5:**
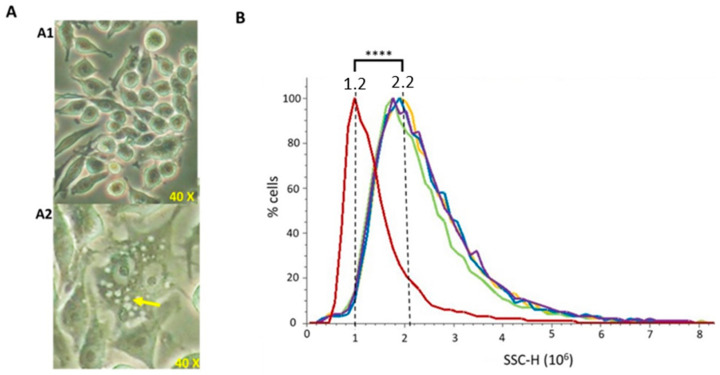
Phagocytosis of outer membrane vesicle naturally released (NT-OMV) or heat treated (HT-OMV) from Enterotoxigenic *Escherichia coli* (ETEC) (ATCC 35401) and collected by ultrafiltration (complete) or ultracentrifugation (sediment) by murine macrophages (RAW 264.7 cells). (**A**) Microscope image of untreated RAW 264.7 cells (A1) and after sediment NT-OMV (20 μg/mL) phagocytosis (A2). Yellow arrow indicates the vesicles phagocytosed by macrophages. (**B**) Untreated RAW 264.7 cells (Red) or treated with sediment or complete OMV (in yellow and green in the graph, respectively) or HT (in blue and purple in the graph, respectively) (20 μg/mL) complexity. On abscissas, there are indicated the relative units of complexity (SSC-H) determined by flow cytometry (**** *p* < 0.0001 vs. untreated cells) (*n* = 3).

To evaluate whether vesicles were able to be phagocytosed by macrophages (which are a model of antigen presenting cells), murine RAW 264.7 cells were incubated with 20 μg/mL of each extract for 24 h. After phagocytosis visual confirmation by optical microscopy (Figure 5A), flow cytometry was used to perform a quantitative comparison among vesicle types. Cells able to uptake the vesicles present higher intracellular complexity (SSC), since higher amounts of vesicles inside the cells produce a higher light dispersion. NT-OMV or HT-OMV complete or sediment from ETEC H10407 were efficiently phagocytosed by macrophages without significant differences between them (Figure 5B).

#### 3.3.4. Cell Stimulation Assay

**Figure 6:**
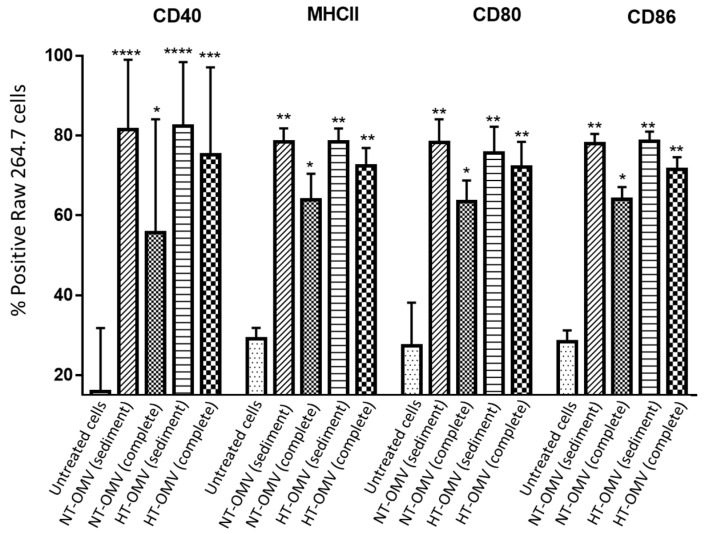
RAW 264.7 cells activation after their incubation with outer membrane vesicles naturally released (NT-OMV) or obtained after heat treatment (HT-OMV) and collected by ultracentrifugation (sediment) or by ultrafiltration (complete) from Enterotoxigenic *Escherichia coli* (ETEC) ATCC 35,401 (1 μg/mL, 37 °C, 5% CO_2_, 24 h). Results are expressed as percentage of positive cells for each marker. (* *p* < 0.05; ** *p* < 0.01; *** *p* < 0.001; **** *p* < 0.0001 vs. untreated cells). Error bars represent standard deviation (*n* = 3).

Once it was demonstrated that the different vesicle types were phagocytosed by macrophages, their capacity of RAW 264.7 cells stimulation was analyzed. To do so, CD40, MHCII, CD80, and CD86 activation and differentiation marker expression was quantified by flow cytometry. Based on the cytotoxicity results, RAW 264.7 cells were incubated with 1 μg/mL of either NT-OMV or HT-OMV sediment or complete vesicles at 37 °C, 5% CO_2_, for 24 h. Results showed that all analyzed vesicles significantly stimulated the expression of the four markers compared to the untreated cells. However, complete NT-OMV induced lower levels of CD40, MHCII, CD80, and CD86 expression (Figure 6 and Figure S2).

**Figure 7:**
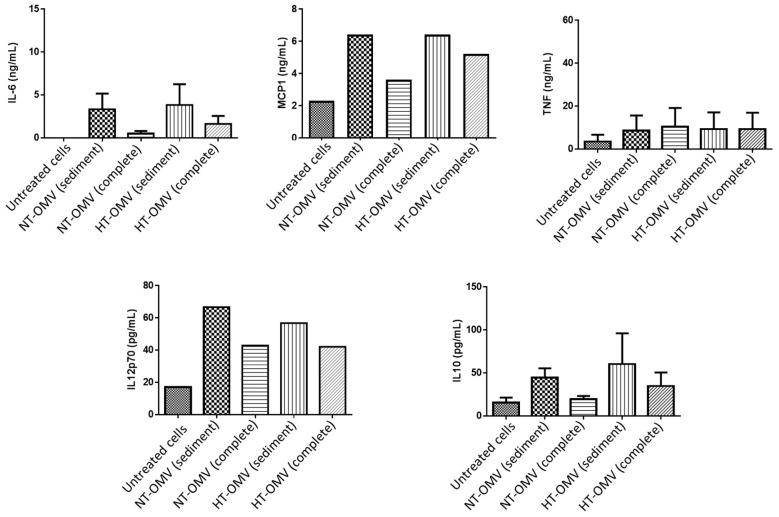
Quantification of IL-6, MCP-1, TNF-α, IL-10, and IL-12p70 cytokines present in activated RAW 264.7 cell supernatants with outer membrane vesicles naturally released (NT-OMV) or obtained after heat treatment (HT-OMV) and collected by ultracentrifugation (sediment) or by ultrafiltration (complete) from Enterotoxigenic *Escherichia coli* (ETEC) ATCC 35,401 (1 μg/mL, 37 °C, 5% CO_2_, 24 h). Cytokine levels were determined by flow cytometry. Results are expressed in ng/mL or pg/mL and untreated cells were used as negative control. Error bars represent standard deviation (*n* = 3).

The activation of macrophages also induces the release of cytokines. Thus, levels of pro-inflammatory (IL-6, MCP-1, TNF-α and IL12p70) and anti-inflammatory (IL-10) cytokines were quantified after cell treatment with 1 μg/mL of each vesicle type at 37 °C, 5% CO_2_, for 24 h. Data from this study indicated that complete vesicles induced lower levels of all cytokines with respect to sediment vesicles. However, these differences were always higher between NT-OMV vesicle types. No significant differences were found between sediment NT-OMV and HT-OMV (Figure 7). 

Taken together, the results indicated that complete NT-OMV shows a diminished capacity to activate macrophages. However, there were no differences between HT-OMV vesicle types. 

### 3.4. Evaluation of Immunogenicity of the Vesicles in BALB/C Mice

**Figure 8:**
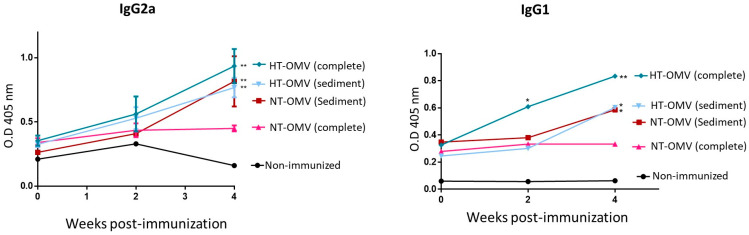
BALB/c mice immunization with outer membrane vesicles naturally released (NT-OMV) or obtained after heat treatment (HT-OMV) and collected by ultracentrifugation (sediment) or by ultrafiltration (complete) from Enterotoxigenic *Escherichia coli* (ETEC) ATCC 35,401 by nasal route. Specific IgG1 and IgG2a serum antibodies levels (1:100 dilution) were determined by ELISA (* *p* < 0.05; ** *p* < 0.01; vs. non-immunized mice). Error bars represent standard deviation (*n* = 3).

To investigate the immunogenicity of the vesicles analyzed in this work, BALB/c mice were immunized with a dose of 25 μg of each vesicle type by nasal route. Four weeks after immunization, mice immunized with complete or sediment HT-OMV showed significantly higher levels of specific IgG2a and IgG1 antibodies compared to non-immunized groups. In this way, sediment NT-OMV also induced significant levels of IgG2a and IgG1 antibodies. In contrast, mice immunized with complete NT-OMV did not show significant IgG2a and IgG1 levels. Thus, the results indicated that the complete HT-OMV vesicle type was the most immunogenic antigenic complex since they induced the highest levels of specific IgG2a and IgG1 antibodies (Figure 8) Thus, the method proposed to produce and purify complete HT-OMV appears to be the most appropriate one.

## 4. Discussion

Currently, there is not a licensed vaccine against ETEC. In this context, we propose the use of ETEC outer membrane vesicles because of their potential as vaccine candidates [12,30]. They present several pathogen-associated molecular patterns (PAMPs) such as lipoproteins or the heat-labile toxin (LT) expressed by ETEC which have immunoadjuvant properties [12,31]. Moreover, bacterial vesicles are able to elicit both systemic and mucosal immunity and they have a safe profile [31]. However, despite the advantages mentioned, their use in vaccination presents an important challenge: the low yield obtained under normal conditions [13]. To improve the release of outer membrane vesicles, we proposed a method based on the heat treatment of bacteria cultures and further isolation of the released vesicles by tangential ultrafiltration, avoiding the expensive ultracentrifugation step. Thus, in the present work, we analyze and compare the cell activation capacity, antigenicity, and immunogenicity of outer membrane vesicles naturally released (NT-OMV) or obtained after heat treatment (HT-OMV) and collected by ultracentrifugation (sediment) or by ultrafiltration (complete) from ETEC ATCC 35401.

Data from vesicle characterization indicated that all vesicle types (NT-OMV and HT-OMV, complete and sediment vesicles) presented a similar LPS pattern and protein profile which includes immunodominant proteins such as OmpA, OmpC, OmpX, OmpW, OmpF, YghJ, Ag43, Skp, lipoproteins, and flagellin, which are demonstrated to boost the immune response elicited by a vaccine [32,33,34,35,36,37,38]. Moreover, ETEC H10407 pathogen-specific proteins are present with similar quantification values in the four bacterial vesicles analyzed in this study, crucial for broad-spectrum ETEC vaccine strategies [9,37,38]. Focusing on LT, this enterotoxin is composed of two subunits: A (enzymatic activity) and B (binding activity). The LT A subunit is responsible for causing a continuous release of water and electrolytes (acute diarrhea) in intestinal enterocytes. Therefore, LT A subunit administration through the oral route (depending on the dose) could provoke the same symptoms as an ETEC infection [31]. It should be noted that the A subunit is not present in the HT-OMV complete fraction. This absence could allow their administration through the oral route, which it is also very interesting in vaccine strategies against enteropathogens. On the other hand, these HT-OMV complete vesicles contain the nontoxic and immunogenic B subunit, which is reported to induce neutralizing antibodies and shows an adjuvant effect [39]. 

Interestingly, these vesicle types demonstrated size variations which could determine the vesicle entry in the cell and the following intracellular signaling route. For instance, Turner et al. studied the entry mechanism of bacterial vesicles isolated from Helicobacter pylori in non-phagocytic cells. They observed that vesicles with average sizes lower than 100 nm preferentially used caveolin-mediated endocytosis, whereas those ranging between 90 and 400 nm entered more efficiently by clathrin- and dynamin-mediated endocytosis. This is an important difference since caveolin-mediated endocytosis is five times slower than clathrin-mediated endocytosis [40]. However, the efficiency of the content release into the cytosol is higher in the first mechanism mentioned [41,42]. Thus, based on this study, it could be supported that the endocytosis and content release into the cytosol should be more efficient for vesicles with sizes lower than 100 nm.

HT-OMV vesicles presented a higher percentage of small vesicles (50–300 nm) compared to NT-OMVs, indicating that they presented the most appropriate size for vaccination purposes.

Furthermore, data indicated that the ultracentrifugation step induced vesicle aggregation compared to complete samples. This phenomenon has been described by other authors [43,44,45,46,47] and some studies propose the use of alternative methods such as filtration [46] or ultrafiltration [47]. Accordingly, complete vesicles are more suitable than those sediments for the purpose of this study.

To determine the cytotoxicity of the samples, RAW 264.7 cells were incubated with each extract and their proliferation was monitored in real time. Results showed that bacterial vesicles did not provoke cellular toxicity for 48 h. Further in vivo studies are needed to determine the real toxicity of the samples analyzed. However, these preliminary data indicate that vaccine use of the bacterial vesicles is safe. Therefore, based on these findings, we proceeded with further cell activation and in vivo studies.

Results from macrophage stimulation studies showed that samples provoked a significant increase in the expression of the molecular markers CD40, MHCII, CD80, and CD86. In addition, they induced the release of pro-inflammatory cytokines such as IL-6 and MCP-1. These data indicated that the extracts analyzed in this work were able to activate macrophages eliciting a Th1 (pro-inflammatory) immune response which is required for host defense against bacterial pathogens [48]. Among the samples, complete HT-OMV elicited the highest levels of macrophage activation, whereas complete NT-OMV induced the lowest activation rate. Furthermore, data obtained from HEK293 activation through TLR4 indicated that complete samples provoke a higher activation of the cells. Since LPS is the main agonist of the receptor TLR4 [49], it could be hypothesized that complete samples induce a higher cell activation due to the presence of higher levels of free LPS or lower vesicle aggregates, resulting in a better interaction between LPS and TLR4. This component, LPS, is endotoxic but also immunogenic. It contributes to vaccine immunogenicity since its interaction with TLR4 induces the release of pro-inflammatory cytokines resulting in the recruitment of macrophages to the luminal side of the intestine. For this reason, several authors consider the inclusion of an adequate dose of LPS in the vaccine as necessary [50,51].

Taking cellular study results together, it can be determined that samples were able to stimulate cells inducing preferentially a pro-inflammatory (pro-Th1) response, which is the typical immune response against ETEC infections [9], and it is necessary to eliminate the pathogen [47]. The Th1 immune response results in specific antibody production at the systemic and mucosal sites preventing ETEC colonization [52]. In this way, clinical studies demonstrated that specific antibodies against CFs (e.g., CFA/I, CS3, or CS21) or adhesins (e.g., EtpA or EatA) were protective against diarrheal disease provoked by ETEC [53]. Therefore, we hypothesize that ETEC vesicles can interact with intestine epithelial and immune cells, inducing a pro-Th1 immune response. 

Finally, BALB/c mice were immunized with NT-OMV or HT-OMV complete or sediment vesicles. Those mice immunized with HT-OMV extracts showed higher levels of specific antibodies IgG1 and IgG2a compared to NT-OMV samples. These differences could be a consequence of several factors: size and composition, among other factors. NT-OMVs are released from specific regions of the bacterial membrane containing specific lipids and proteins, whereas HT-OMV release is forced from different regions of the membrane [10]. Thus, we hypothesized that NT-OMVs could be enriched in virulence factors which are less immunostimulatory, potentially enabling them to evade the host immune system. In contrast, HT-OMV vesicles could present more quantity and more varied PAMPs. Moreover, NT-OMVs showed more aggregates and larger vesicles which demonstrated less capability to induce an immune response in the murine model. On the contrary, the in vivo study showed that the less aggregated and smaller complete HT-OMV vesicles should be more efficient to enter the cell and to induce a pro-inflammatory immune response. Furthermore, BALB/c mice immunized with sediment NT-OMV showed higher specific antibodies compared to complete NT-OMV. These data were in accordance with the results of cellular activation assays and could suggest that the ultracentrifugation step eliminates one or more inhibitory components. In contrast, there were no differences between the HT-OMV sample’s immunogenicity, indicating that the inhibitory components could be inactivated by heat treatment. 

In conclusion, complete HT-OMV vesicles showed the most appropriate size for vaccination, they activated macrophages and HEK293-TLR4 cells, and they elicited the highest levels of specific IgG1 and IgG2a serum antibodies in BALB/c mice. Therefore, it can be concluded that the method to produce and isolate complete HT-OMV was the more adequate one for the purpose of this work. The heat treatment of the cultures is an important advantage since this treatment inactivates the bacteria allowing further processing of the cultures under safety conditions [54]. In addition, the production and isolation of the complete sample of HT-OMV vesicles is another advantage since ultracentrifugation is a complex process which requires specialized personnel and expensive equipment and its maintenance [55]. Consequently, the elimination of this step results in a cost-effective method and a cheaper vaccine. This is of the utmost importance since ETEC infections and disease mainly affects middle- and low-income countries; consequently, the vaccine must be accessible to these regions [37]. Therefore, this study provides an optimized method for ETEC bacterial vesicles production and isolation, providing the basis for the development of a cost-effective and broadly protective ETEC vaccine. Further studies will be needed to evaluate in vivo toxicity, the bacterial vesicle’s ability to elicit specific antibodies not only to the whole vesicle, but also specific to each pathogen-specific virulence factor, and to evaluate their protective efficacy.

## Figures and Tables

**Table 1:** Distribution of vesicle size of the analyzed samples: NT-OMV (sediment), NT-OMV (complete), HT-OMV (sediment), and HT-OMV (complete). The distribution is expressed in percentage (%). Data expressed as mean ± S.D. (*n* = 3).

Vesicle Type	Vesicles in PBS (%)			Sonicated Vesicles in PBS-Tween (%)		
	<50 nm	50–300 nm	>300 nm	<50 nm	50–300 nm	>300 nm
NT-OMV ^a^ (sediment)	0.0 ± 0.0	41.3 ± 2.8	58.7 ± 2.8	32.0 ± 0.0	23.7 ± 0.6	44.3 ± 0.6
NT-OMV ^a^ (complete)	23.3 ± 12.5	38.7 ± 1.5	38 ± 14.0	35.7 ± 0.6	30.3 ± 0.6	34 ± 0.5
HT-OMV ^b^ (sediment)	9.3 ± 2.0	44.3 ± 2.5	46.3 ± 0.6	16.0 ± 8.4	60.0 ± 11.3	24 ± 9.1
HT-OMV ^b^ (complete)	24.0 ± 0.0	44.5 ± 0.7	31.5 ± 0.7	22.5 ± 2.1	59.5 ± 4.9	18 ± 7.0

a, outer-membrane vesicles naturally released (NT-OMV), b, outer-membrane vesicles obtained after heat treatment of the bacterial cultures (HT-OMV).

**Table 2:** **Major *Enterobacteriaceae* proteins** identified in the outer membrane vesicles (NT-OMV) (sediment and complete fraction) and in the vesicles obtained after heat treatment (HT-OMV) (sediment and complete fractions) isolated from the ETEC H10407 strain (ATCC 35401). Protein quantification is expressed in Log iBaqs.

			*Log* iBaqs.			
Uniprot Identifier	Gene	Protein	NT-OMV (Sediment)	NT-OMV (Complete)	HT-OMV (Sediment)	HT-OMV (Complete)
E3PJ90	*yghJ ETEC_3241*	Putative lipoprotein YghJ	8.04	8.33	6.39	5.43
E3PD73	*flu ETEC_4462*	Putative antigen 43 (Fluffing protein)	6.82	7.41	7.87	7.42
D3H0H9	*ompA EC042_1042*	Outer membrane protein A	6.54	7.28	6.45	7.19
D3H0Q5	*ompC EC042_2456*	Outer membrane protein C	8.64	7.70	9.05	6.59
E3PI44	*ETEC_0881*	Outer membrane protein X	8.64	9.06	9.53	9.35
E3PL14	*ETEC_1358*	Outer membrane protein W	8.36	8.40	8.78	8.70
E3PIV0	*ETEC_0997*	Outer membrane protein F	7.54	7.98	8.13	7.31
E3PAU9	*ETEC_2032*	Flagellin	8.88	8.84	8.49	8.77
E3PDD0	*ETEC_0173*	Chaperone protein Skp	7.40	7.46	8.62	8.23
E3PBA3	*dnaK ETEC_0013*	Chaperone protein DnaK	8.79	7.95	7.88	8.63
E3PDA1	*groEL ETEC_4490*	Chaperonin GroEL (60 kDa chaperonin) (Cpn60)	8.99	9.18	7.31	7.96
E3PNC3	*ETEC_1771*	Osmotically inducible lipoprotein E	7.92	7.92	9.32	8.94
E3PIP9	*ETEC_3168*	Putative lipoprotein	7.72	8.28	8.16	8.00
E3PMQ2	*lpp ETEC_1710*	Major outer membrane lipoprotein Lpp (Braun lipoprotein)	6.97	6.97	7.94	7.50
E3PKJ6	*lolB ETEC_1313*	Outer-membrane lipoprotein LolB	6.79	7.24	8.62	7.98
B7UTL1	*traV E2348_P1_065*	Pilus assembly lipoprotein	7.25	7.98	7.25	6.99
D3GZW1	*lolA EC042_0983*	Outer-membrane lipoprotein carrier protein	5.89	6.03	7.32	7.45
E3PDA9	*ETEC_4498*	Outer membrane lipoprotein Blc	6.64	7.15	8.06	7.59

**Table 3:** **Enterotoxigenic *Escherichia coli* (ETEC) pathogen-specific virulence factors** identified in the outer membrane vesicles (NT-OMV) (sediment and complete fraction) and in the vesicles obtained after heat treatment (HT-OMV) (sediment and complete fractions) isolated from the ETEC H10407 strain (ATCC 35401). Protein quantification is expressed in Log iBaqs.

			*Log* iBaqs.			
Uniprot Identifier	Gene	Protein	NT-OMV (Sediment)	NT-OMV (Complete)	HT-OMV (Sediment)	HT-OMV (Complete)
E3PP99	*etpA ETEC_p948_0110*	Two-partner secreted adhesin EtpA	7.32	8.11	7.68	7.11
E3PPA0	*etpB ETEC_p948_0120*	Putative two-partner secretion transporter EtpB	6.51	7.42	7.29	6.74
Q9XD84	*tibA ETEC_2141*	Adhesin/invasin TibA autotransporter	7.71	8.17	8.07	7.69
Q84GK0	*eatA ETEC_p948_0020*	Serine protease EatA	7.54	7.50	7.47	7.30
A7ZGL8	*eltA EcE24377A_F0020*	Heat-labile enterotoxin A chain	7.12	7.82	5.94	0.00
D7GK42	*eltB ETEC1392/75_p1018_007*	Heat-labile enterotoxin B chain	7.76	8.58	7.24	6.51
D0Z6V6	*relB ETEC_p666_0010*	Antitoxin of toxin-antitoxin stability system	0.00	0.00	6.21	7.69
E3PPC3	*cfaA ETEC_p948_0390*	CfA/I fimbrial subunit A	5.77	6.25	6.74	5.64
E3PPC4	*cfaB ETEC_p948_0400*	CFA/I fimbrial subunit B	8.47	8.53	6.77	7.26
E3PPC5	*cfaC ETEC_p948_0410*	Cfa/I fimbrial subunit C	4.64	5.83	6.13	4.62
E3PPC6	*cfaE ETEC_p948_0420*	Cfa/I fimbrial subunit E	5.83	6.42	6.18	5.29

## Data Availability

No new data were created or analysed during this study. Data sharing is not applicable to this article.

## Supplementary Materials

The following supporting information can be downloaded at: https://www.mdpi.com/article/10.3390/microorganisms11082088/s1, Figure S1: LPS pattern of the membrane vesicle naturally released (NT-OMV) or heat treated (HT-OMV) from Enterotoxigenic *Escherichia coli* (ETEC) (ATCC 35401) that were collected by ultrafiltration (complete) or ultracentrifugation (sediment). Figure S2: RAW 264.7 cells activation after their incubation with outer membrane vesicles naturally released (NT-OMV) or obtained after heat treatment (HT-OMV) and collected by ultracentrifugation (sediment) or by ultrafiltration (complete) from Enterotoxigenic *Escherichia coli* (ETEC) ATCC 35401.
