# Supplementary material for: Optimization of Enterotoxigenic Escherichia coli (ETEC) Outer Membrane Vesicles Production and Isolation Method for Vaccination Purposes
Source: Microorganisms. 2023 Aug 15;11(8):2088. doi: 10.3390/microorganisms11082088 (PMC10458947; doi:10.3390/microorganisms11082088)
Supplement: Supplementary file 1 [file microorganisms-11-02088-s001.zip › microorganisms-2540291-SI.pdf]

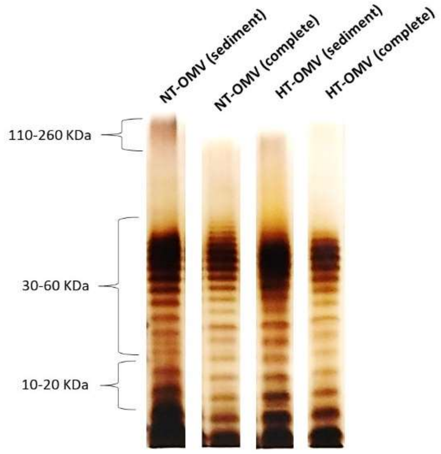

Figure S1. LPS pattern of the membrane vesicle naturally released (NT-OMV) or heat-treated (HT-OMV) from Enterotoxigenic *Escherichia coli* (ETEC) (ATCC 35401) that were collected by ultrafiltration (complete) or ultracentrifugation (sediment). SDS-PAGE with Silver staining of each sample. Molecular weight markers are indicated on the left in kDa, indicating the bands corresponding to short (10-20 KDa), long (30-60 kDa) and very long (120-260 kDa) antigen O chains.

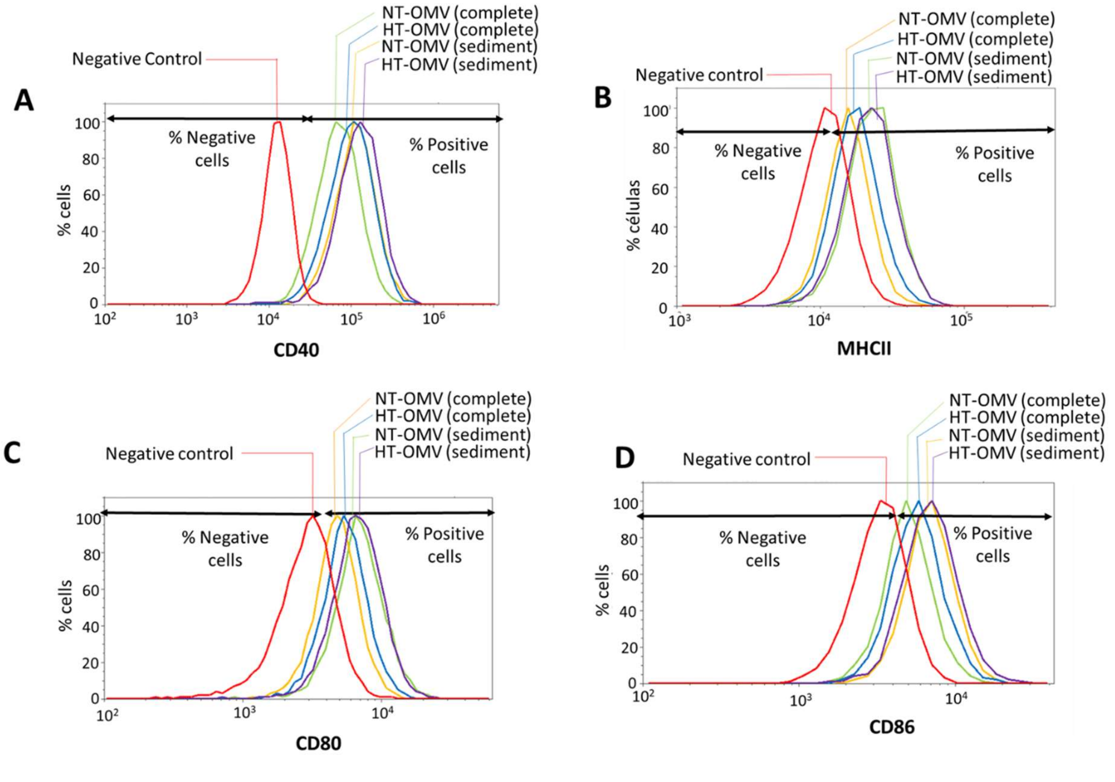

Figure S2. Raw 264.7 cells activation after their incubation with outer membrane vesicles naturally released (NT-OMV) or obtained after heat-treatment (HT-OMV) and collected by ultracentrifugation (sediment) or by ultrafiltration (complete) from Enterotoxigenic *Escherichia coli* (ETEC) ATCC 35401 (1 µg/mL, 37 ºC, 5% CO_2, 24 h). **A-D**. Graphs show the intensity of the fluorescence emitted by Raw 264.7 cells treated with each sample (abscissa axis) and it is indicated the value from cells were considered positive for each marker: CD40 (A), MHCII (B), CD80 (C) or CD86 (D).
